# Eosinophilic Colitis and *Clostridioides difficile* Sepsis With Rapid Remission After Antimicrobial Treatment; A Rare Coincidence and Its Pathogenic Implications

**DOI:** 10.3389/fmed.2020.00328

**Published:** 2020-07-21

**Authors:** Simona Alexandra Iacob, Mihaela Cristina Olariu, Diana Gabriela Iacob

**Affiliations:** ^1^Infectious Diseases Department, Carol Davila University of Medicine and Pharmacy, Bucharest, Romania; ^2^Infectious Diseases Department, The National Institute of Infectious Diseases “Matei Bals”, Bucharest, Romania; ^3^Gastroenterology Department, Carol Davila University of Medicine and Pharmacy, Bucharest, Romania

**Keywords:** eosinophilic colitis, *Clostridioides difficile*, intestinal inflammation, dysbiosis, sepsis, pathogenesis, intestinal eosinophilic infiltrate, hypereosinophilia

## Abstract

Eosinophilic colitis is a rare inflammatory disorder of the digestive tract with chronic evolution and unknown pathophysiological mechanisms. The article describes the case of a 64-year old woman with a history of asthma and hypereosinophilia, who presented to a surgical department for persistent abdominal pain in the past 4 months, weight loss and malabsorption. She was diagnosed with eosinophilic colitis based on the colonoscopic result indicating extensive eosinophilic infiltration of the colonic mucosa correlated with the laboratory data and abdominal CT scan results. Following the colonoscopy, the patient developed fever, hypotension and diarrhea and was transferred to an Infectious Diseases Department with a presumptive diagnosis of abdominal sepsis. Treatment with ertapenem was immediately started. Metronidazole was also added due to a PCR positive stool test for *Clostridioides difficile* toxins encoding-genes. The patient displayed a rapid remission of the fever and of the intestinal complaints following antibiotic therapy and was discharged after 14 days. During a 3 months follow-up, the patient remained asymptomatic with normal values of laboratory parameters except for a persistent hypereosinophilia. The case outlines two distinguishing features: a histopathologic diagnosis of eosinophilic colitis, a rare diagnosis of a patient with chronic abdominal pain and an unexpected and rapid remission of the eosinophilic colitis following the antibiotic treatment and the restoration of the intestinal eubiosis.

## Background

Eosinophils are specialized granulocytes able to coordinate multiple pathogenic pathways involved in the allergic, inflammatory and antimicrobial response. Eosinophils arise in the bone marrow and travel to mucosal tissues, where they regulate the local immune response and tissue repair processes (1). In the gastrointestinal tract, most eosinophils reside in the lamina propria of the stomach and intestines (2). Under inflammatory conditions, eosinophils accumulate in gut-associated lymphoid tissue (3) where they promote immune homeostasis (4) and play an active role against helminths and pathogenic bacteria including *Clostridioides difficile (C. difficile)* intestinal invasion (5–9). On the other hand, significant eosinophilic infiltrates of the gastrointestinal mucosa were described in several conditions, including eosinophilic gastrointestinal diseases and to a lesser extend in patients with inflammatory bowel disease (10) or in those who gradually evolve toward inflammatory bowel diseases (11–13). Eosinophilic gastrointestinal diseases comprise a group of rare diseases with a controversial pathogenesis and therapeutic armamentarium. Depending on the location of the eosinophilic infiltrate, eosinophilic gastrointestinal diseases include esophageal, gastric and intestinal disorders. The number of documented eosinophilic gastrointestinal diseases cases is still low, with an estimated prevalence of around 3.3–5.1/100,000 persons (14, 15).

Hence, most data on eosinophilic gastrointestinal diseases derives from only a few hundred case reports (16–22). Of these, eosinophilic colitis appear to be a particularly rare entity with a challenging diagnosis (23, 24). Currently, only small case series or single case reports of eosinophilic colitis were reported in literature (14, 15, 17, 25–28).

We present the clinical course of a patient with symptomatic eosinophilic colitis who developed nosocomial *C. difficile* infection and sepsis following the diagnostic colonoscopic procedure. The patient displayed an unexpectedly rapid and favorable evolution of these severe infections but also a longstanding improvement of the eosinophilic colitis flare after prompt antibiotic therapy. The article further discusses current literature data on the immunomodulatory role of eosinophils and presents a viewpoint regarding its pathogenic significance in the intestinal inflammatory response in *C. difficile* infection and dysbiosis.

## Case Presentation

**Figure 1:**
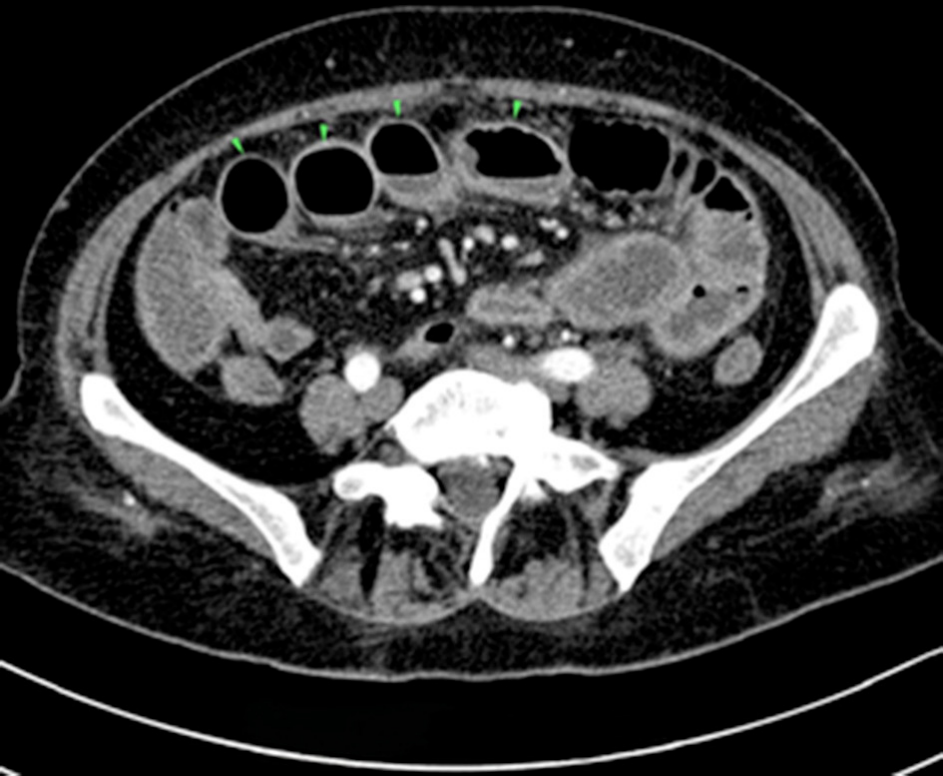
Computed tomography of the abdomen and pelvis. Dilated ileal loops (arrowheads).

A 64-year old lady was hospitalized in a surgical department for abdominal pain lasting for more than 4 months and accompanied by vomiting, flatulence, fatigue and a 10 kg weight loss, without fever or chills. The patient had a history of asthma with recurrent exacerbations, and negative allergological assessments and had received inhalatory steroids for the past 3-years. The thoracic and abdominal CT examination disclosed diffuse intestinal wall thickening and dilated intestinal loops, sigmoid diverticulosis and a small pleural and pericardial effusion (Figure 1, Supplementary Figure 1). Superior endoscopy revealed a minimal erythematous gastritis and the colonoscopic evaluation confirmed sigmoid diverticulosis and segmental colitis. Five biopsy specimens were collected for examination during the colonoscopy. No specific yellow nodules, pseudomembranes or other lesions suggestive for *C. difficile* infection were observed during the colonoscopy. Following this procedure, the patient suddenly developed fever and vomiting. The abdominal pain gradually aggravated and the patient developed a severe diarrhea. Given the high probability of an abdominal sepsis, the patient was transferred to the Department of Infectious Diseases.

**Figure 2:**
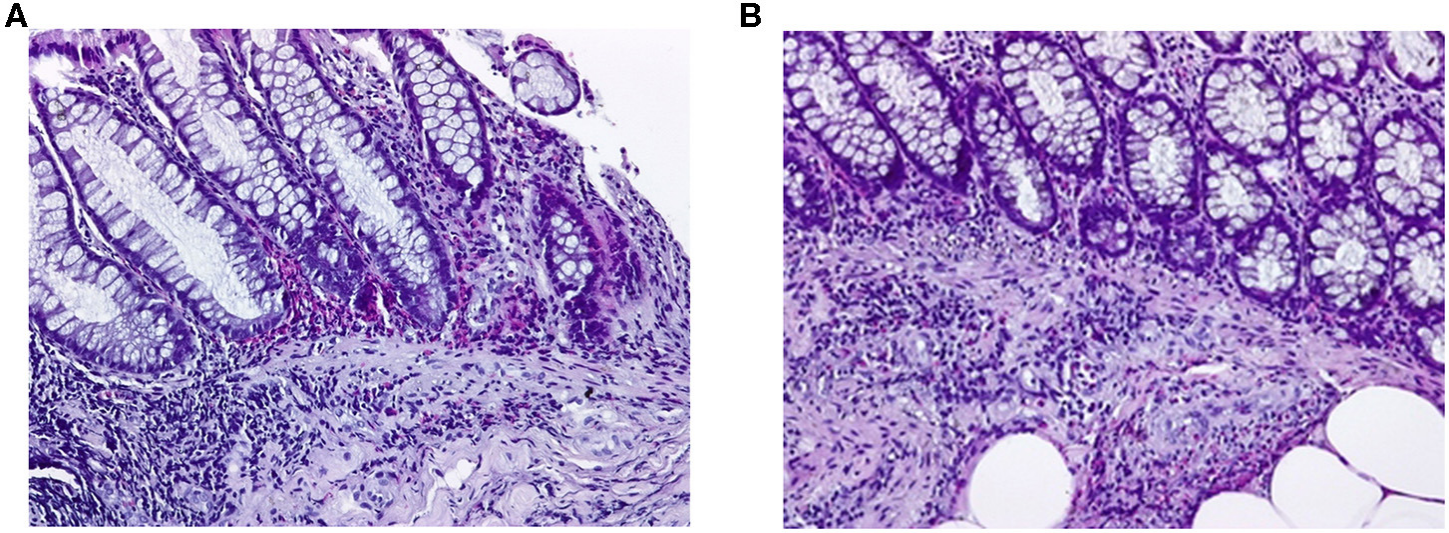
Eosinophilic colitis, histological section. Photography with objective at **200X**. Biopsy was fixed in formalin, embedded in paraffin and stained with hematoxylin-eosin. **(A)** Colonic mucosa with numerous eosinophils infiltrating the lamina propria, of which some with an intraepithelial localization. Polymorphonuclear leukocytes and eosinophils are also seen infiltrating the muscularis mucosa. **(B)** Numerous eosinophils infiltrating the colonic submucosa.

On admission the patient was febrile (39°C), pale, hypotensive (80/60 mmHg), tachycardic (heart rate 95/min) with a distended painful abdomen, peripheral edema and watery stools. Laboratory parameters displayed an intense inflammatory syndrome, increased liver enzymes, positive procalcitonin, hypoalbuminemia, and anemia secondary to the chronic malabsorption but also hypereosinophilia (twice the normal values) and increased IgE levels (three times higher than the normal values) (Table 1). *C. difficile* infection was suspected on the PCR stool assay for toxigenic genes of *C. difficile* (GeneXpert). Blood, stool and urine cultures remained negative. The result of the colonic biopsy was retrieved 48 h after admission and showed a significant eosinophilic infiltrate in the colonic mucosa and submucosa, as well as vasculitis, with numerous eosinophils in the vascular wall. The bioptic specimens did not show any signs of granulomas or malignancy and the histopathologic examination thus pleaded for eosinophilic colitis (Figures 2A,B). Considering the histopathological analysis along with the laboratory and imaging findings, the current presentation was considered as a case of eosinophilic colitis complicated with *C. difficile* infection and sepsis. The patient was initially treated as an abdominal sepsis with ertapenem 1 g/day, with the clinical remission of the fever and the improvement of the laboratory parameters, including the procalcitonin. After receiving the positive results of the PCR stool assay, the patient also received metronidazol 250 mg every 6 h and continued ertapanem for 14 days with a rapid favorable outcome. Upon discharge she was afebrile, asymptomatic, with decreasing IgE levels and normal values of the inflammatory markers. Corticoid treatment was not used during the hospitalization or on discharge. Three months after discharge the patient remained asymptomatic, with no digestive symptoms or asthma exacerbations and normal laboratory values except for persistent hypereosinophilia. The patient also exhibited increasing values of the eosinophils in the following 6 months, raising concerns of a malignant proliferation. She was then admitted to a hematology unit and later to a gastroenterology ward and was placed on corticosteroid treatment with favorable results. On follow-up the patient remained asymptomatic, with no relapse within the following 3-years. The eosinophil count gradually decreased, yet remained at the upper normal limit until now.

**Table 1:** Laboratory investigations in a case of eosinophilic colitis complicated with *C. difficile* infection and sepsis.

**Laboratory findings**	**Reference range**	**Day 1**	**Day 3**	**Day 5**	**Day 7**	**Day 90**	**Six months later**	**Three-years later**
White blood cells (cells/mm^3^)	3.9–9.6	9.1	10.3	9.5	7.9	10	20.38	9.85
Neutrophil granulocytes (cells/mm^3^)	1.4–6.5	5.1	3.53	3.7	3.8	6.5	12.02	4.37
Eosinophils (cells/mm^3^)	0–0.7	1.8	3.39	3.2	1.95	2.2	2.59	0.81
Lymphocytes (cells/mm^3^)	1.2–3.4	0.9	1.86	1.7	1.6	0.8	4.41	3.88
Hemoglobin g/dL	12–17.2	8.6	7.8	8.5	8.5	12	11.4	13.3
Thrombocytes (cells/mm^3^)	200–400	397	442	480	532	483	478	328
Erythrocyte sedimentation rate (mm/h)	<36	110	100	70	36	16	48	
Fibrinogen (mg/dL)	200–393	782	750	494		320		
C-reactive protein (mg/L)	0–3	280					16.9	
Procalcitonin (ng/mL)	Absent	0.65	0.50	0.14	<0.05	0		
Ig E (UI/mL)	0–295	956	–	–	–	300	1,356	
IgG (g/L)	7–16	12.2						
IgM (g/L)	0.4–2.3	0.85						
IgA (g/L)	2.78	2.78						
Complement C3/C4 (g/L)	0.7–1.75/ 0.15–1.8	1.11/0.3						
Hepatitis B, C, HIV tests		Negative					Negative	
ALT (U/L)	9–52	41					57	
Lipase (U/L)	23–300	297						
Glucose (mg/Dl)	65–105	97					84	
Creatinine (mg/Dl)	0.7–1.2	0.7					0.84	
Serum albumin (g/Dl)	3.5–4.1	1.74	1.60	2.07	2.53	4.1	3.4	
Blood culture (aerobic/anaerobic), Uroculture, Stool culture^*^		Negative						
Stool microscopic examination including stool for ova or parasites		Negative for ova, parasites, leucocytes, yeast and molds	Negative		Negative		Negative	
Stool culture for Strongyloides stercoralis							Negative	
Toxoplasma gondii IgG							Negative	
Toxocara canis IgG							Negative	
Trichinella spirallis IgG							Negative	
C difficile tcdA and tcdB genes (stool, PCR, GeneXpert)		Positive						
Food alergens (IgE detection)		Negative					Negative	
Serum autoimmunity markers (Eneasystem III test)^**^and rheumatoid factor		Negative					Negative	
Bone marrow aspiration (6 months later)	Cytologic smear: 42–44% eosinophils, with cells in all stages of maturation							
Bone marrow biopsy	Myeloid hyperplasia with 50–55% eosinophils; no *cellular atypia*							
(6 months later)	Immunohistochemical tests: CD34 positivity <5% (isolate cells); CD68/PG-M1 positivity (only isolate monocytes); tryptase expression positive only in a few mast cells; CD3 positivity in rare *small* T lymphocytes							
	Genetic tests: *BCR-ABL1* gene sequence: negative (multiplex PCR)							
	*FIP1L1-PDGFRA* and *PDGFRB* fusion gene: negative expression							
	Conclusion. There are no specific *immunohistochemistry* or genetic aberrancies for eosinophilia-associated myeloproliferative neoplasms							

* *Stool culture (Shigella, Salmonella, Yersinia, enteroinvasive, E coli serotypes)*;

** *ANA Screen, dsDNA, ssDNA, Anti SS-A (Ro),Anti-SS-B (La), Anti Sm, Anti Jo, Anti SCL-70, AntiCentromer, p-ANCA, cANCA, Antiglyadin IgA, IgG, Antitransglutaminase IgA, IgG, Mitochondrial antigen AMA- (Eneasystem III test)*.

## Discussion

Eosinophilic colitis is a rare and insufficiently characterized disease. The clinical presentation of eosinophilic colitis is highly unspecific and prompts a very wide differential diagnosis, which includes inflammatory bowel diseases, celiac diseases, neoplasia, vasculitis, the hypereosinophilic syndrome, drug induced colitis or various infectious etiologies (28). Currently, the only definitive criteria for eosinophilic colitis is the finding of an extensive eosinophilic infiltrate of the intestinal wall, containing at least 30 eosinophils per high-power field in at least five high-power fields associated with endoscopic abnormalities (29, 30). However, the histopathologic criteria are not standardized and differ between studies (22, 23, 31–33). Thus, in the absence of characteristic clinical and laboratory findings, many patients could remain undiagnosed (23). The underlying pathophysiologic mechanisms in eosinophilic colitis have not been fully elucidated and the therapeutic options are limited. Allergic mechanisms appear to play an important, albeit unknown role in a large number of patients and consequently the use of corticosteroids in severe cases is considered beneficial (16, 18, 30). An atopic state has been reported by various authors in 41.8% of patients with eosinophilic colitis (14). However, its presence is not mandatory (18, 34) and IgE–mediated mechanisms appear to be more common in young patients compared to adults (35). Peripheral eosinophilia was observed in 74–83.3% of cases with eosinophilic colitis and has been associated with a high rate of relapse (18, 20, 26). Still, serum eosinophilia was not recorded in all cases. Various authors have underlined the absence of a correlation between serum eosinophilia and the colitis outcome or the intestinal eosinophilic infiltrate, thus suggesting the secondary role of eosinophilia in the onset of colitis (36, 37).

Clinical and experimental studies have showed that the trafficking of eosinophils to the gut and their activation is stimulated by intestinal signals released by the intestinal epithelia, as well as by type 2 innate lymphoid cells (ILC2) or by mucosal immune response (38–40). Hence, murine studies showed that eosinophils normally accumulate in the lamina propria of the gut and stomach and their presence is mainly regulated by eotaxin-1, a chemokine released by epithelial cells (38, 40). Eotaxin expressed throughout the gastrointestinal tract displays a synergism with IL-5 and IL-13, two ILC2- secreted cytokines (39, 40). Other potent regulators of tissue eosinophilia and ILC2 activity include the cytokines IL-25 (a member of the IL-17 family) and IL-33 (a member of the IL-1 family), two central mediators of the Th2 immunity and Foxp3(+) regulatory T (Treg) cells immunosuppressive activities of the gut barrier (41, 42). Thus, the intestinal trafficking of eosinophils is a result of the signals released by the intestinal barrier and of the immune mediators regulated by various physiologic or pathologic stimuli.

### The Immunomodulatory Role of Eosinophils in the Intestinal Inflammatory Response

The ILC2-eosinophil axis plays a major role in the specific anti-inflammatory response to allergen exposure as well as in the protective immunity against pathogens and the remodeling and intestinal repair (43–45). Eosinophils constitutively express various toll like receptors which enable the recognition of numerous microbial antigens, sustain secretory IgA production, dendritic cells activation and Th1/Th17 pro-inflammatory response as well as the retention of immunosuppressive Treg cells in the lamina propria (4, 46, 47). Thus, eosinophils display both a pro-inflammatory and a cytotoxic potential against pathogenic germs, while also supporting a protective role in gut homeostasis. In studies on healthy mice, intestinal eosinophils preserve the integrity of the epithelial barrier and also regulate the innate host defense and tissue remodeling (4, 48–51).

Nevertheless, ILC2-Th2 secreted cytokines and intestinal eosinophils have been linked with significant histopathological changes during eosinophilic esophagitis or other intestinal disorders (2, 44, 52, 53). Still, there is insufficient data regarding the conditions which facilitate this phenotypical switch of eosinophils and which enable their extensive and chronic infiltration of the colonic wall. The changes of the intestinal environment and the pathologic transformation of tissue eosinophils have been associated with multiple factors such as a genetic predisposition, various intestinal allergens, as well as with persistent intestinal infections or severe and prolonged dysbiosis (54–57). Nevertheless, the pathologic role of intestinal eosinophils during dysbiosis or enteral infections such as *C. difficile* remains controversial (58–60).

### Intestinal Eosinophils and *C. difficile* Infection

The number of cases with *C. difficile* infection-associated eosinophilic colitis is extremely small. Kim et al. published a similar case (61). None of the existing prevalence studies have approached the impact of the intestinal eosinophilic infiltrate on the colonization with *C. difficile* strains. However, the role of intestinal eosinophils in the attenuation of the inflammatory immune response during *C. difficile* infection was underlined by Buonomo and other authors and was attributed to the mucosal eosinophilia (6, 62–64) and IL-25 induction. Additionally the previous studies also discussed the role of IL-33, in the recruitment of eosinophils (65) and ILC2 activation (45), in the maintenance of intestinal eubiosis (50) or in the reduction of *C. difficile* mortality (45). In this respect, most murine models highlighted the ability of IL-33 to orchestrate the inflammatory response and tissue remodeling, further leading to a subsequent decrease in the translocation of pathogenic bacteria (66–68) and to the preservation of the intestinal homeostasis (66, 68). Hence, the IL-25/IL-33/ILC2 axis activation and gut eosinophils regulate the local immune response during acute enteral infections and play a specific regulatory role in inflammatory response during *C. difficile* infections (45). At the same time, IL-33 remains a contradictory cytokine with a marked pro-inflammatory potential (69, 70). Hence, a high and persistent level of IL-33 could contribute to an excessive eosinophilic response and to the build- up of pathogenic lesions. Nevertheless, the previous hypotheses require further clinical validation and if proven could significantly change the current perspective on the role played by intestinal eosinophils in the pathogenesis of intestinal inflammatory diseases.

### Intestinal Eosinophils and Microbiota Homeostasis

The microbiota plays a remarkable anti-infectious role, enabling the survival of commensal germs and the elimination of pathogens as a result of its connections with the intestinal immunity and the intestinal epithelia. The immune-microbiota homeostasis is closely linked to the eosinophilic immune response, explaining the rapid activation of the latter during dysbiosis (4). Regarding the underlying pathogenic mechanisms, both enteral infections and related dysbiosis increase the concentration of intestinal eosinophils through a Th17- pro-inflammatory response, as well as through the ILC2-eosinophil axis which ensues after the epithelial release of IL-25/IL-33 cytokines (71).

Once stimulated, the intestinal eosinophils exert an important immunomodulatory role, regulating the excessive inflammatory response (72). This regulatory role involves the suppression of the Th17 response elicited by IL-1 receptor antagonist IL-1 Ra, the activation of CD103+ dendritic cell activation, the differentiation of Treg cells and the synthesis of secretory antimicrobial IgA (47, 50, 73, 74). Eosinophils are thus involved in the preservation of the intestinal homeostasis and are correlated with the composition of the intestinal microbiota, as shown experimentally by Chu et al. (4) and Jung et al. (50).

Chu et al. (4) analyzed the microbiota of eosinophil-deficient mice and recorded various significant differences, most of which concerned segmented filamentous bacteria, a member of Clostridiales with an essential role in the Th17 induction of and T cell response (75, 76). At the same time, Jung et al. (50) showed that the lack of gastrointestinal eosinophils reduces the level of secretory IgAs and decreases the ensuing protection against microbial pathogens, leading to dysbiosis. These experiments support the ***L***ocal ***I***mmunity ***A***nd/or ***R***emodeling/***R***epair (LIAR) hypothesis (51) formulated by Lee in 2010 according to which in a Th1/Th17 polarized microenvironment, the intestinal eosinophilic infiltrate is a regulatory and not a destructive event, an effect, rather than a cause of the intestinal inflammation. Hence, its presence in a severe illness (10) could be an adaptation mechanism to antigenic exposure meant to attenuate the Th17 immune response and to favor tissue regeneration.

### Notable Findings of the Clinical Case

The case discussed above displayed several notable features regarding its diagnosis and evolution.

For one thing, the patient reported a non-specific onset which initially suggested an abdominal tumor, given the diffuse abdominal pain and the accompanying weight loss in the past 4 months However, the subsequent histopathological exam disclosed numerous eosinophils located in the lamina propria of colonic mucosa and muscularis mucosa, accompanied by hypereosinophilia and an increased IgE serum level, which supported the final diagnosis of eosinophilic colitis. Furthermore, the disease was considered severe based on the extension of the eosinophilic infiltrates, the prolonged diseases course and hypoalbuminemia and anemia secondary to the colonic malabsorption (36).

Secondly, the diarrheal syndrome was interpreted in the context of a *C. difficile* infection, according to the case definitions elaborated by the Infectious Diseases Society of America (IDSA) and European Society of Clinical Microbiology and Infectious Diseases (ESCMID). The diagnosis of *C. difficile* infection was based on the following arguments: (a) the sudden debut of a diarrheal syndrome following colonoscopy; (b) the positive PCR assay indicating *C. difficile* toxin genes *tcdA* and *tcdB;* (c) the absence of other causes explaining the diarrheal syndrome (Table 1); (d) the therapeutic response to oral vancomycin.

Regarding the risk of colonization with toxigenic strains of *C. difficile*, while this alternative is possible in asymptomatic patients, we argue that this is less likely given the sudden development of a diarrheic syndrome in our patient in the absence of other causes (Table 1) or of known risk factors for *C. difficile* colonization (77, 78).

Data regarding the risk of colonization with toxigenic strains of C. difficile remains controversial. While no data are available on *C. difficile* colonization in patients with eosinophilic colitis, Cowardin et al. showed in a mouse model of *C. difficile* colitis that toxigenic strains inhibit the protective eosinophilic response through a TLR2 mediated mechanism and favor the inflammatory response and the development of diarrhea (62). Nevertheless, the hypothesis of a toxigenic colonization before colonoscopy cannot be disproved. Given the preexistent inflammatory lesions it is probable that such a colonization would have induced a previous episode of *C. difficile* infection (78) which did not occur.

The colonoscopy also exposed the patient to supplementary complications. The risk of *C. difficile* infection after colonoscopy has been previously suggested (79, 80) and it is considered a rare occurrence. Nosocomial *C. difficile* strains exhibit high cytotoxicity and higher rates of recurrence and bloodstream infections (81). The occurrence of *C. difficile* infection in cases of inflammatory bowel disease leads to the activation of pro-inflammatory cytokines, including the IL-1β/Th17 axis (78) which aggravates the course of diseases (82–85), increases the risk of *C. difficile* recurrences (84) and raises the mortality by four times (86). Similarly the risk of sepsis in *C. difficile* infection is a severe event due to the altered intestinal barrier and the subsequent bacterial translocation (87, 88).

Nevertheless, in our case the patient displayed a favorable and unexpectedly rapid evolution, along with the significant improvement of the symptoms of eosinophilic colitis following antibiotic treatment.

The evolution of our case is compatible with a protective role of eosinophils toward *C. difficile*-associated infection as suggested by Buonomo et al. (6) and Cowardin et al. (62) as well as a beneficial role of eosinophils in gut homeostasis discussed by Jung et al. (50) and Chu et al. (4).

Our case also underlines the role of dysbiosis and the impact of its treatment on the flare of eosinophilic colitis. The clinical outcome of the patient suggested that the intestinal dysbiosis remitted; had the dysbiosis persisted it would have aggravated under ertapenem and the infection with *C. difficile* would have recurred. The rapid improvement without a recurrence was particularly intriguing: an antibiotic treatment in a patient with eosinophilic colitis resolved both the *C. difficile* infection and alleviated the manifestations of eosinophilic colitis. In other words, if the gut equilibrium between pathogens and commensals is re-established, the clinical course of intestinal inflammatory diseases should also improve, as has been shown by Khan et al. (89). Hence, it is possible that this finding could also apply to eosinophilic colitis, an intestinal inflammatory disease.

It is probable that the chronic inflammatory response in the eosinophilic colitis is maintained through either occult intestinal infections and related dysbiosis or through an abberant immune response to the intestinal microbiota, two key events which could favor the intestinal acummulation of eosinophils. Should this be the case, it is probable that the administration of synbiotics, fecal transplantation or other regulatory therapeutic agents of microbiota could address both the inflammatory response and dysbiosis. Hence, Dai and colleagues reported a severe case of eosinophilic gastroenteritis, with a rapid remission following fecal microbiota transplantation and glucocorticoid treatment (90). However, the role of dysbiosis in the exacerbation of the intestinal immune response and in the pathogenesis of intestinal inflammation has already been shown in experimental studies (91), in patients with inflammatory bowel disease (92, 93) and in various murine experiments (94, 95). This finding has led to an increased interest for therapeutic fecal transplantation in inflammatory bowel disease (96–98), although its indication has not been formally studied in eosinophilic colitis (99). Additional research is needed to establish the impact of therapeutic strategies against dysbiosis on the clinical outcome of eosinophilic colitis.

### The Significance of the Case

The study of gut dysbiosis in eosinophilic colitis was not previously approached although similar correlations have been observed in other intestinal inflammatory syndromes (100, 101). The importance of this case lies in a clinical hypothesis to be proven by future studies, regarding the correlation between eosinophilic colitis and intestinal dysbiosis. At the same time, we wanted to highlight that current results from experimental data support the physiopathological hypothesis of eosinophils as defense cell-lines attracted to the intestinal mucosa in order to maintain intestinal homeostasis. Hence, the presence of eosinophils in the gut indicates a previous intestinal “conflict,” potentially due to a shift of the harbored intestinal flora. If proven, this concept could enable a more simple, less expensive and less aggressive treatment which could potentially be implemented in eosinophilic colitis as well as in other intestinal inflammatory diseases. The publication of other cases could be useful for the understanding of other physio-pathological aspects in eosinophilic syndromes.

## Conclusion

The current case describes an uncommon gastrointestinal disorder- eosinophilic colitis—complicated by nosocomial *C. difficile* infection and severe sepsis following a diagnostic colonoscopy. Moreover, the rapid and intriguing improvement of colitis after antibiotic treatment underlines the relevance of restoring the intestinal eubiosis in order to achieve the remission of both the infectious complications and the flare of eosinophilic colitis.

The case additionally highlights the role of eosinophils in the protection of the intestinal barrier and intestinal immune response during *C. difficile* infection and reveals how the reestablishment of the microbiota homeostasis leads to a favorable evolution of eosinophilic colitis.

## Data Availability Statement

All datasets generated for this study are included in the article/Supplementary Material.

## Ethics Statement

Written informed consent was obtained from the patient's representative for the publication of this case report.

## Author Contributions

SI wrote the manuscript. DI collected the patient's clinical data, searched literature data, and critically reviewed the manuscript. MO collected the imagistic data and critically reviewed the manuscript. All authors contributed to the article and approved the submitted version.

## Conflict of Interest

The authors declare that the research was conducted in the absence of any commercial or financial relationships that could be construed as a potential conflict of interest.

## Supplementary Material

Supplementary Figure 1 Computed tomography of the abdomen and pelvis. Mild circumferential and diffuse intestinal wall thickening with parietal enhancement (arrows).

## References

[1] Travers J Rothenberg ME. Eosinophils in mucosal immune responses. Mucosal Immunol. (2015) 8:464–75. 10.1038/mi.2015.2 25807184 PMC4476057

[2] Jung Y Rothenberg ME. Roles and regulation of gastrointestinal eosinophils in immunity and disease. J Immunol. (2014) 193:999–1005. 10.4049/jimmunol.1400413 25049430 PMC4109658

[3] Mishra A Hogan SP Brandt EB Rothenberg ME. Peyer's patch eosinophils: identification, characterization, and regulation by mucosal allergen exposure, interleukin-5, and eotaxin. Blood. (2000) 96:1538–44. 10.1182/blood.V96.4.1538 10942403

[4] Chu VT Beller A Rausch S Strandmark J Zänker M Arbach O. Eosinophils promote generation and maintenance of immunoglobulin-a-expressing plasma cells and contribute to gut immune homeostasis. Immunity. (2014) 40:582–93. 10.1016/j.immuni.2014.02.014 24745334

[5] Hogan SP Waddell A Fulkerson PC. Eosinophils in infection and intestinal immunity. Curr Opin Gastroenterol. (2013) 29:7–14. 10.1097/MOG.0b013e32835ab29a 23132211 PMC3924957

[6] Buonomo EL Cowardin CA Wilson MG Saleh MM Pramoonjago P Petri WA. Microbiota-regulated IL-25 increases eosinophil number to provide protection during *Clostridium difficile* infection. Cell Rep. (2016) 16:432–43. 10.1016/j.celrep.2016.06.007 27346351 PMC4945404

[7] Yousefi S Gold JA Andina N Lee JJ Kelly AM Kozlowski E. Catapult-like release of mitochondrial DNA by eosinophils contributes to antibacterial defense. Nat Med. (2008) 14:949–53. 10.1038/nm.1855 18690244

[8] Svensson L Wennerås C. Human eosinophils selectively recognize and become activated by bacteria belonging to different taxonomic groups. Microbes Infect. (2005) 7:720–8. 10.1016/j.micinf.2005.01.010 15857806

[9] Ramirez GA Yacoub M-R Ripa M Mannina D Cariddi A Saporiti N. Eosinophils from physiology to disease: a comprehensive review. Biomed Res Int. (2018) 2018:9095275. 10.1155/2018/9095275 29619379 PMC5829361

[10] Bischoff SC Mayer J Nguyen Q-T Stolte M Manns MP Immunohistological assessment of intestinal eosinophil activation in patients with eosinophilic gastroenteritis and inflammatory Bowel disease. Am J Gastroenterol. (1999) 94:3521–9. 10.1111/j.1572-0241.1999.01641.x 10606314

[11] Uzunismail H Hatemi I Dogusoy G Akin O. Dense eosinophilic infiltration of the mucosa preceding ulcerative colitis and mimicking eosinophilic colitis: report of two cases. Turk J Gastroenterol. (2006) 17:53–7. Available online at: https://www.turkjgastroenterol.org/en/dense-eosinophilic-infiltration-of-the-mucosa-preceding-ulcerative-colitis-and-mimicking-eosinophilic-colitis-report-of-two-cases-1621539 16830279

[12] Mutalib M Blackstock S Evans V Huggett B Chadokufa S Kiparissi F. Eosinophilic gastrointestinal disease and inflammatory bowel disease in children. Eur J Gastroenterol Hepatol. (2015) 27:20–3. 10.1097/MEG.0000000000000230 25358014

[13] Chetcuti Zammit S Cachia M Sapiano K Gauci J Montefort S Ellul P. Eosinophilic gastrointestinal disorder: is it what it seems to be? Ann Gastroenterol. (2018) 31:475–9. 10.20524/aog.2018.0263 29991893 PMC6033761

[14] Jensen ET Martin CF Kappelman MD Dellon ES. Prevalence of eosinophilic gastritis, gastroenteritis, and colitis: estimates from a national administrative database. J Pediatr Gastroenterol Nutr. (2016) 62:36–42. 10.1097/MPG.0000000000000865 25988554 PMC4654708

[15] Mansoor E Saleh MA Cooper GS. Prevalence of eosinophilic gastroenteritis and colitis in a population-based study, from 2012 to 2017. Clin Gastroenterol Hepatol. (2017) 15:1733–41. 10.1016/j.cgh.2017.05.050 28603057

[16] Reed C Woosley JT Dellon ES. Clinical characteristics, treatment outcomes, and resource utilization in children and adults with eosinophilic gastroenteritis. Dig Liver Dis. (2015) 47:197. 10.1016/j.dld.2014.11.009 25547198 PMC4339627

[17] Díaz del Arco C Taxonera C Olivares D Fernández Aceñero MJ. Eosinophilic colitis: case series and literature review. Pathol Res Pract. (2018) 214:100–4. 10.1016/j.prp.2017.09.029 29103770

[18] Zhang L Duan L Ding S Lu J Jin Z Cui R. Eosinophilic gastroenteritis: clinical manifestations and morphological characteristics, a retrospective study of 42 patients. Scand J Gastroenterol. (2011) 46:1074–80. 10.3109/00365521.2011.579998 21623674

[19] Vithayasai N Jennuvat S Lertsatit A. Eosinophilic gastrointestinal disease: analysis of sixteen cases from ten years experience in Thailand. J Med Assoc Thai. (2011) 94(Suppl. 3):S41–8. 22043753

[20] Pineton de Chambrun G Gonzalez F Canva J-Y Gonzalez S Houssin L Desreumaux P. Natural history of eosinophilic gastroenteritis. Clin Gastroenterol Hepatol. (2011) 9:950–6.e1. 10.1016/j.cgh.2011.07.017 Available online at: https://www.researchgate.net/publication/51761537_Eosinophilic_gastrointestinal_disease_analysis_of_sixteen_cases_from_ten_years_experience_in_Thailand 21806952

[21] Lee CM Changchien CS Chen PC Lin DY Sheen IS Wang CS . Eosinophilic gastroenteritis: 10 years experience. Am J Gastroenterol. (1993) 88:70–4. 8420276

[22] Abassa K-K Lin X-Y Xuan J-Y Zhou H-X Guo Y-W. Diagnosis of eosinophilic gastroenteritis is easily missed. World J Gastroenterol. (2017) 23:3556. 10.3748/wjg.v23.i19.3556 28596692 PMC5442092

[23] Turner KO Sinkre RA Neumann WL Genta RM. Primary colonic eosinophilia and eosinophilic colitis in adults. Am J Surg Pathol. (2017) 41:225–33. 10.1097/PAS.0000000000000760 27792062

[24] Hentschel F Jansen AF Günther M Pauli R Lüth S. Eosinophil counts in mucosal biopsies of the ileum and colon: interobserver variance affects diagnostic accuracy. Patholog Res Int. (2018) 2018:1–7. 10.1155/2018/2638258 30519390 PMC6241360

[25] Pesek RD Reed CC Muir AB Fulkerson PC Menard-Katcher C Falk GW. Increasing rates of diagnosis, substantial co-occurrence, and variable treatment patterns of eosinophilic gastritis, gastroenteritis, and colitis based on 10-year data across a multicenter consortium. Am J Gastroenterol. (2019) 114:984–94. 10.14309/ajg.0000000000000228 31008735 PMC6554065

[26] Alfadda AA Shaffer EA Urbanski SJ Storr MA. Eosinophilic colitis is a sporadic self-limited disease of middle-aged people: a population-based study. Colorectal Dis. (2014) 16:123–9. 10.1111/codi.12464 24138295

[27] Alfadda AA Storr MA Shaffer EA. Eosinophilic colitis: an update on pathophysiology and treatment. Br Med Bull. (2011) 100:59–72. 10.1093/bmb/ldr045 22012125

[28] Egan M Furuta GT. Eosinophilic gastrointestinal diseases beyond eosinophilic esophagitis. Ann Allergy Asthma Immunol. (2018) 121:162–7. 10.1016/j.anai.2018.06.013 29940308

[29] Lucendo AJ Arias A. Eosinophilic gastroenteritis: an update. Expert Rev Gastroenterol Hepatol. (2012) 6:591–601. 10.1586/egh.12.42 23061710

[30] Sunkara T Rawla P Yarlagadda KS Gaduputi V. Eosinophilic gastroenteritis: diagnosis and clinical perspectives. Clin Exp Gastroenterol. (2019) Volume 12:239–53. 10.2147/CEG.S173130 31239747 PMC6556468

[31] Bates AWH Diagnosing eosinophilic colitis: histopathological pattern or nosological entity? Scientifica. (2012) 2012:9 10.6064/2012/682576 PMC3820477 24278727

[32] Collins MH Histopathologic features of eosinophilic esophagitis and eosinophilic gastrointestinal diseases. Gastroenterol Clin North Am. (2014) 43:257–68. 10.1016/j.gtc.2014.02.007 24813514

[33] Lowichik A Weinberg AG. A quantitative evaluation of mucosal eosinophils in the pediatric gastrointestinal tract. Mod Pathol. (1996) 9:110–4. 8657715

[34] Gonsalves N. Food allergies and eosinophilic gastrointestinal illness. Gastroenterol Clin North Am. (2007) 36:75–91. 10.1016/j.gtc.2007.01.003 17472876

[35] Furuta GT Forbes D Boey C Dupont C Putnam P Roy S Eosinophilic gastrointestinal diseases. J Pediatr Gastroenterol Nutr. (2008) 47:234–8. 10.1097/MPG.0b013e318181b1c3 18664881

[36] Talley NJ Shorter RG Phillips SF Zinsmeister AR. Eosinophilic gastroenteritis: a clinicopathological study of patients with disease of the mucosa, muscle layer, and subserosal tissues. Gut. (1990) 31:54–8. 10.1136/gut.31.1.54 2318432 PMC1378340

[37] Kamal MF Shaker K Jaser N Leimoon BA. Eosinophilic gastroenteritis with no peripheral eosinophilia. Ann Chir Gynaecol. (1985) 74:98–100. 4026181

[38] Matthews AN Friend DS Zimmermann N Sarafi MN Luster AD Pearlman E. Eotaxin is required for the baseline level of tissue eosinophils. Proc Natl Acad Sci USA. (1998) 95:6273. 10.1073/pnas.95.11.6273 9600955 PMC27654

[39] Nussbaum JC Van Dyken SJ von Moltke J Cheng LE Mohapatra A Molofsky AB. Type 2 innate lymphoid cells control eosinophil homeostasis. Nature. (2013) 502:245–8. 10.1038/nature12526 24037376 PMC3795960

[40] Mishra A Hogan SP Lee JJ Foster PS Rothenberg ME. Fundamental signals that regulate eosinophil homing to the gastrointestinal tract. J Clin Invest. (1999) 103:1719–27. 10.1172/JCI6560 10377178 PMC408388

[41] Fort MM Cheung J Yen D Li J Zurawski SM Lo S. IL-25 induces IL-4, IL-5, and IL-13 and Th2-associated pathologies *in vivo*. Immunity. (2001) 15:985–95. 10.1016/S1074-7613(01)00243-6 11754819

[42] Schmitz J Owyang A Oldham E Song Y Murphy E McClanahan TK IL-33, an interleukin-1-like cytokine that signals via the IL-1 receptor-related protein ST2 and induces T helper type 2-associated cytokines. Immunity. (2005) 23:479–90. 10.1016/j.immuni.2005.09.015 16286016

[43] Klose CSN Artis D. Innate lymphoid cells as regulators of immunity, inflammation and tissue homeostasis. Nat Immunol. (2016) 17:765–74. 10.1038/ni.3489 27328006

[44] Yazdani R Sharifi M Shirvan AS Azizi G Ganjalikhani-Hakemi M. Characteristics of innate lymphoid cells and their role in immunological disorders. Cell Immunol. (2015) 298:66–76. 10.1016/j.cellimm.2015.09.006 26429626

[45] Frisbee AL Saleh MM Young MK Leslie JL Simpson ME Abhyankar MM. IL-33 drives group 2 innate lymphoid cell-mediated protection during *Clostridium difficile* infection. Nat Commun. (2019) 10:2712. 10.1038/s41467-019-10733-9 31221971 PMC6586630

[46] Loktionov A. Eosinophils in the gastrointestinal tract and their role in the pathogenesis of major colorectal disorders. World J Gastroenterol. (2019) 25:3503–26. 10.3748/wjg.v25.i27.3503 31367153 PMC6658389

[47] Chu DK Jimenez-Saiz R Verschoor CP Walker TD Goncharova S Llop-Guevara A. Indigenous enteric eosinophils control DCs to initiate a primary Th2 immune response *in vivo*. J Exp Med. (2014) 211:1657–72. 10.1084/jem.20131800 25071163 PMC4113937

[48] Lampinen M Rönnblom A Amin K Kristjansson G Rorsman F Sangfelt P. Eosinophil granulocytes are activated during the remission phase of ulcerative colitis. Gut. (2005) 54:1714–20. 10.1136/gut.2005.066423 15886302 PMC1774808

[49] Furuta GT Nieuwenhuis EES Karhausen J Gleich G Blumberg RS Lee JJ. Eosinophils alter colonic epithelial barrier function: role for major basic protein. Am J Physiol Gastrointest Liver Physiol. (2005) 289:G890–7. 10.1152/ajpgi.00015.2005 16227527

[50] Jung Y Wen T Mingler MK Caldwell JM Wang YH Chaplin DD. IL-1β in eosinophil-mediated small intestinal homeostasis and IgA production. Mucosal Immunol. (2015) 8:930–42. 10.1038/mi.2014.123 25563499 PMC4481137

[51] Lee JJ Jacobsen EA McGarry MP Schleimer RP Lee NA. Eosinophils in health and disease: the LIAR hypothesis. Clin Exp Allergy. (2010) 40:563–75. 10.1111/j.1365-2222.2010.03484.x 20447076 PMC2951476

[52] Hogan SP Mishra A Brandt EB Royalty MP Pope SM Zimmermann N. A pathological function for eotaxin and eosinophils in eosinophilic gastrointestinal inflammation. Nat Immunol. (2001) 2:353–60. 10.1038/86365 11276207

[53] Doherty TA Baum R Newbury RO Yang T Dohil R Aquino M. Group 2 innate lymphocytes are enriched in active eosinophilic esophagitis. J Allergy Clin Immunol. (2015) 136:792–4.e3. 10.1016/j.jaci.2015.05.048 26233928 PMC4562810

[54] Verma AK Kandikattu HK Manohar M Shukla A Upparahalli Venkateshaiah S Zhu X. Intestinal overexpression of IL−18 promotes eosinophils-mediated allergic disorders. Immunology. (2019) 157:110–21. 10.1111/imm.13051 30779114 PMC6526631

[55] Keely S Walker MM Marks E Talley NJ. Immune dysregulation in the functional gastrointestinal disorders. Eur J Clin Invest. (2015) 45:1350–9. 10.1111/eci.12548 26444549

[56] Walker MM. Inflammation, genetics, dysbiosis, and the environment. J Clin Gastroenterol. (2016) 50:S4–5. 10.1097/MCG.0000000000000613 27622361

[57] Walker MM Potter M Talley NJ. Eosinophilic gastroenteritis and other eosinophilic gut diseases distal to the oesophagus. Lancet Gastroenterol Hepatol. (2018) 3:271–80. 10.1016/S2468-1253(18)30005-0 29533199

[58] Zaiss MM Maslowski KM Mosconi I Guenat N Marsland BJ Harris NL. IL-1β suppresses innate IL-25 and IL-33 production and maintains helminth chronicity. PLoS Pathog. (2013) 9:e1003531. 10.1371/journal.ppat.1003531 23935505 PMC3731249

[59] Villani A-C Lemire M Fortin G Louis E Silverberg MS Collette C. Common variants in the NLRP3 region contribute to Crohn's disease susceptibility. Nat Genet. (2009) 41:71–6. 10.1038/ng.285 19098911 PMC2728932

[60] Alhallaf R Agha Z Miller CM Robertson AAB Sotillo J Croese J. The NLRP3 inflammasome suppresses protective immunity to gastrointestinal helminth infection. Cell Rep. (2018) 23:1085–98. 10.1016/j.celrep.2018.03.097 29694887

[61] Kim TG Park J Seo EH Joo HR Park SH Kim TO Esosinophilic gastroenteritis with *Clostridium difficile*-associated colitis: a case report. Korean J Gastrointest Endosc. (2011) 43:64–8. Available online at: https://www.e-ce.org/upload/pdf/Kjge043-01-16.pdf

[62] Cowardin CA Buonomo EL Saleh MM Wilson MG Burgess SL Kuehne SA. The binary toxin CDT enhances *Clostridium difficile* virulence by suppressing protective colonic eosinophilia. Nat Microbiol. (2016) 1:16108. 10.1038/nmicrobiol.2016.108 27573114 PMC5010011

[63] Hosoki K Nakamura A Nagao M Hiraguchi Y Tokuda R Wada H. Differential activation of eosinophils by ‘probiotic' *Bifidobacterium bifidum* and ‘pathogenic' *Clostridium difficile*. Int Arch Allergy Immunol. (2010) 152:83–9. 10.1159/000312131 20523069

[64] Crook DW Walker AS Kean Y Weiss K Cornely OA Miller MA. Fidaxomicin versus vancomycin for *Clostridium difficile* infection: meta-analysis of pivotal randomized controlled trials. Clin Infect Dis. (2012) 55(Suppl. 2):S93–103. 10.1093/cid/cis499 22752871 PMC3388031

[65] Cherry WB Yoon J Bartemes KR Iijima K Kita H. A novel IL-1 family cytokine, IL-33, potently activates human eosinophils. J Allergy Clin Immunol. (2008) 121:1484–90. 10.1016/j.jaci.2008.04.005 18539196 PMC2821937

[66] Williams MA O'Callaghan A Corr SC. IL-33 and IL-18 in inflammatory bowel disease etiology and microbial interactions. Front Immunol. (2019) 10:1091. 10.3389/fimmu.2019.01091 31139196 PMC6527769

[67] Malik A Sharma D Zhu Q Karki R Guy CS Vogel P. IL-33 regulates the IgA-microbiota axis to restrain IL-1α-dependent colitis and tumorigenesis. J Clin Invest. (2016) 126:4469–81. 10.1172/JCI88625 27775548 PMC5127671

[68] Gro P Doser K Falk W Obermeier F Hofmann C. IL-33 attenuates development and perpetuation of chronic intestinal inflammation. Inflamm Bowel Dis. (2012) 18:1900–9. 10.1002/ibd.22900 22508383

[69] McEntee CP Finlay CM Lavelle EC. Divergent roles for the IL-1 family in gastrointestinal homeostasis and inflammation. Front Immunol. (2019) 10:1266. 10.3389/fimmu.2019.01266 31231388 PMC6568214

[70] Masterson JC Capocelli KE Hosford L Biette K McNamee EN de Zoeten EF. Eosinophils and IL-33 perpetuate chronic inflammation and fibrosis in a pediatric population with stricturing Crohn's ileitis. Inflamm Bowel Dis. (2015) 21:2429–40. 10.1097/MIB.0000000000000512 26218140 PMC4567482

[71] Dias PM Banerjee G. The role of Th17/IL-17 on eosinophilic inflammation. J Autoimmun. (2013) 40:9–20. 10.1016/j.jaut.2012.07.004 22906357

[72] Marichal T Mesnil C Bureau F. Homeostatic eosinophils: characteristics and functions. Front Med. (2017) 4:101. 10.3389/fmed.2017.00101 28744457 PMC5504169

[73] Sugawara R Lee E-J Jang MS Jeun E-J Hong C-P Kim J-H. Small intestinal eosinophils regulate Th17 cells by producing IL-1 receptor antagonist. J Exp Med. (2016) 213:555–67. 10.1084/jem.20141388 26951334 PMC4821642

[74] Chen H-H Sun A-H Ojcius DM Hu W-L Ge Y-M Lin X. Eosinophils from murine lamina propria induce differentiation of naïve T cells into regulatory T cells via TGF-β1 and retinoic acid. PLoS ONE. (2015) 10:e0142881. 10.1371/journal.pone.0142881 26587591 PMC4654556

[75] Goto Y Panea C Nakato G Cebula A Lee C Diez MG. Segmented filamentous bacteria antigens presented by intestinal dendritic cells drive mucosal Th17 cell differentiation. Immunity. (2014) 40:594–607. 10.1016/j.immuni.2014.03.005 24684957 PMC4084624

[76] Gaboriau-Routhiau V Rakotobe S Lécuyer E Mulder I Lan A Bridonneau C. The key role of segmented filamentous bacteria in the coordinated maturation of gut helper T cell responses. Immunity. (2009) 31:677–89. 10.1016/j.immuni.2009.08.020 19833089

[77] Hung Y-P Tsai P-J Hung K-H Liu H-C Lee C-I Lin H-J Impact of toxigenic *Clostridium difficile* colonization and infection among hospitalized adults at a district hospital in southern Taiwan. PLoS ONE. (2012) 7:e42415 10.1371/journal.pone.0042415 22876321 PMC3411658

[78] Crobach MJT Vernon JJ Loo VG Kong LY Péchiné S Wilcox MH. Understanding Clostridium difficile colonization. Clin Microbiol Rev. (2018) 31: e00021–17. 10.1128/CMR.00021-17 29540433 PMC5967689

[79] Rutala WA Gergen MF Weber DJ. Inactivation of *Clostridium difficile* spores by disinfectants. Infect Control Hosp Epidemiol. (1993) 14:36–9. 10.1086/646628 8432966

[80] Kovaleva J Peters FTM van der Mei HC Degener JE. Transmission of infection by flexible gastrointestinal endoscopy and bronchoscopy. Clin Microbiol Rev. (2013) 26:231–54. 10.1128/CMR.00085-12 23554415 PMC3623380

[81] Xu Q Chen Y Gu S Lv T Zheng B Shen P Hospital-acquired *Clostridium difficile* infection in Mainland China: a seven-year (2009–2016) retrospective study in a large university hospital. Sci Rep. (2017) 7:9645 10.1038/s41598-017-09961-0 28852010 PMC5575102

[82] Saleh MM Frisbee AL Leslie JL Buonomo EL Cowardin CA Ma JZ. Colitis-induced Th17 cells increase the risk for severe subsequent *Clostridium difficile* infection. Cell Host Microbe. (2019) 25:756–65.e5. 10.1016/j.chom.2019.03.003 31003940 PMC6509008

[83] Li Y Xu H Xu T Xiao M Tang H Wu D Case–control study of inflammatory bowel disease patients with and without *Clostridium difficile* infection and poor outcomes in patients coinfected with *C. difficile* and cytomegalovirus. Dig Dis Sci. (2018) 63:3074–83. 10.1007/s10620-018-5230-1 30094621 PMC6182452

[84] Issa M Vijayapal A Graham MB Beaulieu DB Otterson MF Lundeen S. Impact of *Clostridium difficile* on inflammatory bowel disease. Clin Gastroenterol Hepatol. (2007) 5:345–51. 10.1016/j.cgh.2006.12.028 17368234

[85] Aletaha N Dadvar Z Salehi B Ketabi Moghadam P Niksirat A Jowkar A. Clinical and pathological features of ulcerative colitis in patients with and without *Clostridium difficile* infection; an observational study. Middle East J Dig Dis. (2018) 11:17–23. 10.15171/mejdd.2018.123 31049178 PMC6488494

[86] Ananthakrishnan AN McGinley EL Binion DG. Excess hospitalisation burden associated with *Clostridium difficile* in patients with inflammatory bowel disease. Gut. (2008) 57:205–10. 10.1136/gut.2007.128231 17905821

[87] Lowenkron SE Waxner J Khullar P Ilowite JS Niederman MS Fein AM. *Clostridium difficile* infection as a cause of severe sepsis. Intensive Care Med. (1996) 22:990–4. 10.1007/BF02044130 8905440

[88] Eckel F Huber W Weiss W Lersch C. Recurrent pseudomembranous colitis as a cause of recurrent severe sepsis. Z Gastroenterol. (2002) 40:255–8. 10.1055/s-2002-25147 11961735

[89] Khan KJ Ullman TA Ford AC Abreu MA Abadir A Marshall JK. Antibiotic therapy in inflammatory bowel disease: a systematic review and meta-analysis. Am J Gastroenterol. (2011) 106:661–73. 10.1038/ajg.2011.72 21407187

[90] Dai Y-X Shi C-B Cui B-T Wang M Ji G-Z Zhang F-M. Fecal microbiota transplantation and prednisone for severe eosinophilic gastroenteritis. World J Gastroenterol. (2014) 20:16368–71. 10.3748/wjg.v20.i43.16368 25473198 PMC4239532

[91] Feng T Wang L Schoeb TR Elson CO Cong Y. Microbiota innate stimulation is a prerequisite for T cell spontaneous proliferation and induction of experimental colitis. J Exp Med. (2010) 207:1321–32. 10.1084/jem.20092253 20498021 PMC2882839

[92] Stepankova R Powrie F Kofronova O Kozakova H Hudcovic T Hrncir T Segmented filamentous bacteria in a defined bacterial cocktail induce intestinal inflammation in SCID mice reconstituted with CD45RBhigh CD4+ T cells. Inflamm Bowel Dis. (2007) 13:1202–11. 10.1002/ibd.20221 17607724

[93] Britton GJ Contijoch EJ Mogno I Vennaro OH Llewellyn SR Ng R. Microbiotas from humans with inflammatory bowel disease alter the balance of gut Th17 and RORγt+ regulatory T cells and exacerbate colitis in mice. Immunity. (2019) 50:212–24.e4. 10.1016/j.immuni.2018.12.015 30650377 PMC6512335

[94] Ivanov II Atarashi K Manel N Brodie EL Shima T Karaoz U. Induction of intestinal Th17 cells by segmented filamentous bacteria. Cell. (2009) 139:485–98. 10.1016/j.cell.2009.09.033 19836068 PMC2796826

[95] Burrello C Giuffrè MR Macandog AD Diaz-Basabe A Cribiù FM Lopez G. Fecal microbiota transplantation controls murine chronic intestinal inflammation by modulating immune cell functions and gut microbiota composition. Cells. (2019) 8:517. 10.3390/cells8060517 31142049 PMC6628315

[96] Moayyedi P Surette MG Kim PT Libertucci J Wolfe M Onischi C Fecal microbiota transplantation induces remission in patients with active ulcerative colitis in a randomized controlled trial. Gastroenterology. (2015) 149:102–9.e6. 10.1053/j.gastro.2015.04.001 25857665

[97] Costello SP Hughes PA Waters O Bryant R V. Vincent AD Blatchford P. Effect of fecal microbiota transplantation on 8-week remission in patients with ulcerative colitis. JAMA. (2019) 321:156. 10.1001/jama.2018.20046 30644982 PMC6439766

[98] Imdad A Nicholson MR Tanner-Smith EE Zackular JP Gomez-Duarte OG Beaulieu DB. Fecal transplantation for treatment of inflammatory bowel disease. Cochrane Database Syst Rev. (2018) 11:CD012774. 10.1002/14651858.CD012774.pub2 30480772 PMC6517295

[99] Cammarota G Ianiro G Tilg H Rajilić-Stojanović M Kump P Satokari R. European consensus conference on faecal microbiota transplantation in clinical practice. Gut. (2017) 66:569–80. 10.1136/gutjnl-2016-313017 28087657 PMC5529972

[100] Kostic AD Xavier RJ Gevers D. The microbiome in inflammatory bowel disease: current status and the future ahead. Gastroenterology. (2014) 146:1489–99. 10.1053/j.gastro.2014.02.009 24560869 PMC4034132

[101] Mirsepasi-Lauridsen HC Vrankx K Engberg J Friis-Møller A Brynskov J Nordgaard-Lassen I. Disease-specific enteric microbiome dysbiosis in inflammatory bowel disease. Front Med. (2018) 5:304. 10.3389/fmed.2018.00304 30525037 PMC6256240

