# Supplementary figures and images for: Eosinophilic Colitis and Clostridioides difficile Sepsis With Rapid Remission After Antimicrobial Treatment; A Rare Coincidence and Its Pathogenic Implications
Source: Front Med (Lausanne). 2020 Jul 21;7:328. doi: 10.3389/fmed.2020.00328 (PMC7396602; doi:10.3389/fmed.2020.00328)

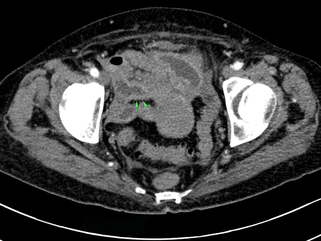

Supplement: Supplementary Figure 1 — Computed tomography of the abdomen and pelvis. Mild circumferential and diffuse intestinal wall thickening with parietal enhancement (arrows). [file Image_1.JPEG]
